# Nicotinamide riboside rescues angiotensin II–induced cerebral small vessel disease in mice

**DOI:** 10.1111/cns.13276

**Published:** 2020-01-14

**Authors:** Cheng‐Cheng Li, Wei‐Xiang Chen, Jie Wang, Min Xia, Zheng‐Cai Jia, Chao Guo, Xiao‐Qin Tang, Ming‐Xi Li, Yi Yin, Xin Liu, Hua Feng

**Affiliations:** ^1^ Department of Neurosurgery Southwest Hospital Third Military Medical University (Army Medical University) Chongqing China; ^2^ State Key Laboratory of Silkworm Genome Biology Southwest University Chongqing China; ^3^ Department of Neurosurgery Southwest Hospital Collaborative Innovation Center for Brain Science Third Military Medical University (Army Medical University) Chongqing China

**Keywords:** angiotensin ‐, arterioles, blood‐brain barrier, cerebral small vessel disease, cognitive impairment, inflammation

## Abstract

**Aims:**

Hypertension is a leading cause of cerebral small vessel disease (CSVD). Currently, treatments for CSVD are limited. Nicotinamide riboside (NR) can protect against vascular injury and cognitive impairment in neurodegenerative diseases. In this study, the protective effects of NR against angiotensin ‐ (Ang ‐)–induced CSVD were evaluated.

**Methods:**

To explore the effects of NR in CSVD, C57BL/6 mice were infused with Ang ‐, and NR was added to the food of the mice for 28 days. Then, short‐term memory, blood‐brain barrier (BBB) integrity, and endothelial function were detected. Arteriole injury and glial activation were also evaluated.

**Results:**

Our data showed that mice infused with Ang ‐ exhibited decreased short‐term memory function and BBB leakage due to decreased claudin‐5 expression and increased caveolae‐mediated endocytosis after 28 days. Furthermore, Ang ‐ decreased the expression of α‐smooth muscle actin (α‐SMA) and increased the expression of proliferating cell nuclear antigen (PCNA) in arterioles and decreased the expression of neurofilament 200 (NF200) and myelin basic protein (MBP) in the white matter. These CSVD‐related damages induced by Ang ‐ were inhibited by NR administration. Moreover, NR administration significantly reduced glial activation around the vessels.

**Conclusion:**

Our results indicated that NR administration alleviated Ang ‐–induced CSVD by protecting BBB integrity, vascular remodeling, neuroinflammation, and white matter injury (WMI)–associated cognitive impairment.

## 1 INTRODUCTION

Cerebral small vessel disease (CSVD), one of the main causes of vascular dementia, accounts for 20% of all dementia and is associated with 25% of stroke.^1^ CSVD affects almost 100% of people older than 90 years and leads to the worsening of Alzheimer's disease (AD) symptoms.^2^ However, there is no reliable treatment for CSVD.^3^, ^4^ CSVD is characterized by white matter hyperintensities, cognitive impairment, cerebral microbleeds, and cerebrovascular injury in patients.^1^, ^5^, ^6^ BBB leakage is considered to be the initiating factor of CSVD and is related to the severity of the disease.^7^

The blood‐brain barrier (BBB) is formed by the endothelium, astroglia, pericytes, and basal lamina.^8^ BBB dysfunction is a widespread phenomenon in patients with CSVD and is associated with impaired cognition.^7^ Impaired cognition is linked to white matter injury (WMI), which can predict the outcome of CSVD in patients.^9^, ^10^ The destruction of the BBB allows harmful substances, such as inflammatory cells and inflammatory factors, to enter the brain parenchyma and then triggers inflammation.^8^ Typical intimal thickening caused by atherosclerosis in areas of hypertensive arterial disease occurs in CSVD,^1^ and CSVD shows stronger associations with vascular inflammation in these areas. Ang ‐ can cause CSVD, including vascular injury, neuroinflammation, and cognitive decline in mice.^11^, ^12^ The application of antihypertension drugs, such as inhibitors of the angiotensin receptor and angiotensin‐converting enzyme, failed to reverse neurological deficits such as cognitive impairment in CSVD patients.^3^, ^13^, ^14^ Alternative treatments for Ang ‐–induced CSVD need to be proposed.

Nicotinamide adenine dinucleotide (NAD+) is a coenzyme that is essential for maintaining the mitochondrial electron transfer chain.^15^ NAD+ is involved in vital intracellular signal transduction, such as cell proliferation, death, and metabolism.^16^ Moreover, nicotinamide riboside (NR) and nicotinamide mononucleotide (NMN), NAD+ precursors, have beneficial effects in neurological degenerative diseases and cardiovascular diseases, such as Parkinson's disease (PD), AD, aging, stroke, and hypertension.^17^, ^18^, ^19^, ^20^, ^21^, ^22^, ^23^ However, the administration of the NAD+ precursors maintains the integrity of BBB, reverses endothelial dysfunction, and improves cognitive function and healthspan.^18^, ^23^, ^24^ The role of NR in CSVD has not been investigated. We hypothesized that NR administration has protective effects against CSVD. Our data illustrated that NR administration improved CSVD, including BBB integrity, neuroinflammation, and cognitive function. NR administration may be a potential therapeutic method for CSVD.

## 2 MATERIALS AND METHODS

### 2.1 Animals

Adult male C57BL/6 mice （8‐10 weeks old, 22‐30 g）were used in this study. The mouse model was induced by using osmotic minipumps (ALZET® Osmotic Pumps, DURECT Corporation) as previously described.^25^ Briefly, the mice were anesthetized with 1% sodium phenobarbital. Then, the hair was removed from the back of the mice, and the skin was disinfected with iodophor and cut with scissors. The osmotic minipumps were subcutaneously implanted and used to administer Ang ‐ (1000 ng/kg/min, purchased from Absin) or saline for 28 days, and the skin was sutured. The mice were divided into three groups: the control, Ang ‐, and NR groups. In the control group, the subcutaneously implanted osmotic pumps contained saline. In the Ang ‐ group, the subcutaneously implanted osmotic pumps contained Ang ‐, and the NR group received chow containing NR (300 mg/g body weight, purchased from AdipoGen Corporation) while also receiving Ang ‐ infusion for 28 days. The experimental procedures were approved by the Laboratory Animal Care and Use Committee of the Army Medical University, China. The mice were maintained in standard housing conditions under a 12‐hour light/dark cycle with free access to food and water.

### 2.2 Immunohistochemistry

Immunohistochemistry was performed according to previously described procedures.^26^ Briefly, anesthetized mice were transcardially perfused with saline, and then, the mice were further perfused with 4% paraformaldehyde (PFA) in phosphate‐buffered saline (PBS). The brains were postfixed in 4% PFA overnight at 4℃ and then dehydrated in 30% sucrose at 4℃ for 2 days. For hematoxylin‐eosin (H&E) staining, the brains were sliced coronally (5 μm) on a vibrating slicer. The sections were incubated in hematoxylin for 2 minutes and eosin for 1 minute. The samples were observed with a BX51 microscope (Olympus) and analyzed with Image‐Pro Plus. For immunofluorescence staining, the brains were embedded in optimal cutting temperature compound (OCT Compound, SAKURA) and then sectioned coronally at a thickness of 30 μm using a cryostat microtome (CM1860UV; Leica). The sections were blocked with 5% bovine serum albumin (BSA) and 0.25% Triton X‐100 for 1.5 hours at room temperature and then incubated with primary antibodies overnight at 4°C, after which the sections were washed with PBS 3 times and incubated with secondary antibodies overnight at 4°C. The following antibodies were used: caveolin‐1 (Cav‐1, rabbit, 1:200; Cell Signaling Technology (CST), s3267), Cav‐1 (mouse, 1:100; Santa Cruz Biotechnology, sc‐53564), α‐SMA (rabbit, 1:200; Abcam, ab7817), MBP (goat, 1:200; Santa Cruz Biotechnology, sc‐13914), PCNA (mouse, 1:100; Santa Cruz Biotechnology, sc‐56), glial fibrillary acidic protein (GFAP) (rabbit 1:200; Abcam, ab7260), and ionized calcium‐binding adapter molecule 1 (IBA1) (goat, 1:100; Santa Cruz Biotechnology, sc20). Alexa Fluor 488–conjugated (mouse, rabbit, 1:1000), Alexa Fluor 555–conjugated (rabbit, 1:1000), and Alexa Fluor 594–conjugated (mouse, 1:1000) secondary antibodies from Invitrogen were used. The nuclei were counterstained with 4′‐6‐diamidino‐2‐phenylindole (DAPI; Santa Cruz Biotechnology). To visualize brain blood vessels, biotinylated *Lycopersicon esculentum* (tomato) lectin (LEL, TL) (1:200; Vector Laboratories, B‐1175) was used to label endothelial cells, and the experimental procedure was the same as that for immunofluorescence. The sections were then incubated with DyLight 488–conjugated Streptavidin (1:200; Vector Laboratories, SA‐5488) overnight at 4°C. The samples were observed with a laser scanning confocal microscope (Zeiss, LSM780) and analyzed by ZEN2012.

### 2.3 BBB integrity assay

The BBB integrity assay was as previously described.^27^ Briefly, dextran （3 kDa, Invitrogen; D3328）was injected into the tail vein after 28 days. Two hours later, anesthetized mice were perfused with saline and 4% PFA, and brain tissues were then sectioned at a thickness of 30 μm for immunohistochemical staining. The sections were stained with lectin. The samples were observed with a laser scanning confocal microscope (Zeiss, LSM780). The fluorescence intensity of dextran was analyzed by using ZEN2012.

### 2.4 Western blot analysis

Western blot method was as previously described.^26^ Briefly, every 100‐mg brain tissue was lysed using lysis solution containing 100 µL RIPA buffer and a mixture of inhibitors (Roche). The protein concentration was determined using a BCA protein assay kit (Beyotime Biotechnology). Brain lysates were diluted in loading buffer solution at 95°C for 5 minutes. Total protein (50 µg) was resolved on 8%‐12% polyacrylamide gels. After gel electrophoresis was completed, the proteins were transferred to polyvinylidene difluoride (PVDF) membranes. The membranes were blocked with 5% nonfat milk in Tris‐buffered saline (TBS) containing 0.1% Tween‐20 for 2 hours at room temperature. Then, the membranes were incubated with primary antibodies overnight at 4°C. After that, the membranes were washed in TBS containing 0.1% Tween‐20 for 3 times and then incubated with horseradish peroxidase–conjugated secondary antibodies for 2 hours at room temperature. The immunoreactive bands were detected using the chemiluminescence reagent kit (Thermo Scientific). The primary antibodies used were as follows: Cav‐1 (rabbit, 1:1000; CST, s3267), ZO‐1 (rabbit 1:1000; Invitrogen, 61‐7300), occludin (rabbit 1:1000; Invitrogen, 71‐1500), claudin‐5 (rabbit 1:1000; Invitrogen, 34‐1600), MBP (goat, 1:1000; Santa Cruz Biotechnology, sc‐13914), GAPDH (mouse, 1:1000; Proteintech, 60004‐1‐lg), TNF‐α (rabbit, 1:1000; Boster BA14901）, and endothelial nitric‐oxide synthase (eNOS) (mouse, 1:1000; Abcam, ab76198). Rabbit HRP‐conjugated, mouse HRP‐conjugated, and goat HRP‐conjugated secondary antibodies were used. Images were taken and analyzed by using Image Lab software (Image Lab 3.0; Bio‐Rad).

### 2.5 Transmission electron microscopy

The Transmission electron microscopy (TEM) method was described in a previous study.^26^, ^27^ Mice were anesthetized with 1% pentobarbital sodium and perfused through the heart with 30 mL saline, followed by 1.25% glutaraldehyde and 2% PFA in 0.1 mol/L phosphate buffer (PB). Then, the brains were rapidly removed and postfixed for 3 days at 4°C. The tissues were washed overnight in 0.1 mol/L sodium cacodylate buffer and then cut in 50‐mm‐thick free‐floating sections using a vibratome. The sections were postfixed with 1% OsO_4_ in PB for 2 hours, counterstained with uranyl acetate, dehydrated in a graded acetone series, infiltrated with propylene oxide, and embedded in Epon. Ultrathin sections (~60 nm) were cut by using an ultramicrotome (LKB‐V, LKB Produkter AB, Bromma) and observed under a transmission electron microscope. To quantify the number of vesicles, the vesicles from 5 random images were chosen for calculation.

### 2.6 Novel object recognition task and target location task

The Novel object recognition (NOR) task and target location task (OLT) were used to assess short‐term recognition memory as previously described.^28^, ^29^ Briefly, the mice underwent environmental adaptation in a 30‐cm × 45‐cm open box for 2 days, and on the third day, the mice were placed in the box with two identical objects for 5 minutes. Then, the mice were placed back in the cage for 15 minutes. The mice were again placed in the box after one object was replaced with a novel object for 5 minutes. The time the mice spent exploring the two objects within 5 minutes was recorded. The Recognition Index (RI, representing the time spent exploring the novel object (T novel) relative to that spent exploring the familiar object (T familiar)) was calculated according to the formula: RI = T novel/(T novel + T familiar). The experimental steps of the OLT were the same as those of the NOR task. Instead of replacing one object with a novel object, we moved the object. Then, we recorded the time that the mice spent exploring the moved object. All the behavioral tests were performed by a blinded investigator.

### 2.7 Statistical analysis

All results are presented as the means ± standard deviations (SDs). Statistical analyses were performed using Prism 6, and *t* test, one‐way analysis of variance (ANOVA), and two‐way ANOVA were used to analyze the data. Significance is indicated as *P < .05, **P < .01, and ***P < .001.

## 3 RESULT

### 3.1 NR administration ameliorates Ang ‐–induced white matter injury and cognitive impairment

**Figure 1:**
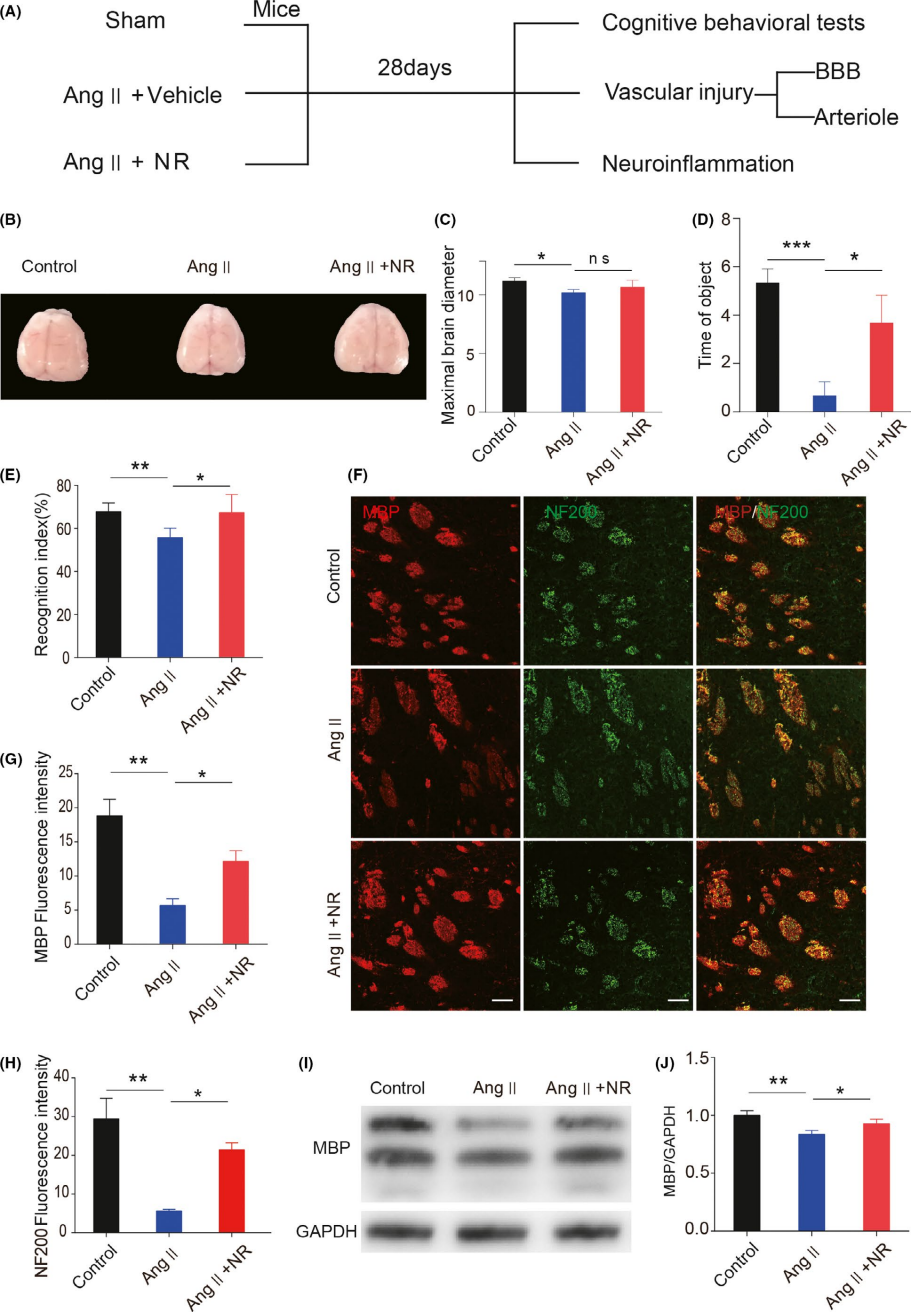
NR administration ameliorates angiotensin ‐–induced myelin degradation and short‐term memory function decline. (A) Experimental design. (B‐C) Representation of brain morphology and the quantification of maximal brain diameter in control, Ang ‐–treated, and NR‐treated mice after 28 d. (D‐E) Recognition memory was measured in the 3 groups by the OLT and NOR task. (F‐H) Representative immunofluorescence images and quantification analysis of NF200 and MBP expression in brain tissues from the 3 groups. (I, J) Representative images of immunoblotting analysis and the quantification of MBP in brain tissues from the 3 groups. The scale bars in F represent 50 μm; *P < .05 and **P < .01, ***P < .001. Ang: angiotensin ‐, Ang + NR: angiotensin ‐+nicotinamide riboside. The results are presented as the means ± standard deviations (SDs)

To examine cognitive function, the maximal diameter of the cerebral was measured. There was a significant decline in the Ang ‐ group compared with the control group, and NR treatment attenuated the Ang ‐–induced decrease in cerebral diameter (control, 11.17 ± 0.16; Ang ‐, 10.17 ± 0.17; NR, 10.67 ± 0.34; Figure 1B,C）. Short‐term memory recognition was assessed by the NOR task and OLT after the mice were infused with Ang ‐ for 28 days. The data showed that cognitive ability was significantly decreased and was rescued by NR administration (OLT: control, 5.33 ± 0.33; Ang ‐, 0.67 ± 0.33; NR, 3.67 ± 0.67; Figure 1D; NOR: control, 67.75 ± 2.03%; Ang ‐, 55.70 ± 2.22%; NR, 67.40 ± 4.172%; Figure 1E). WMI was common in CSVD and was associated with cognitive decline. The mice infused with Ang ‐ showed decreased immunofluorescence intensities of MBP and NF200. NR administration significantly increased the intensities of MBP and NF200 compared with those in the Ang ‐ infusion group（Figure 1F‐H）. Western blot result showed that NR treatment restored the decreased expression of MBP caused by Ang ‐ infusion（control, 1.000 ± 0.023; Ang ‐, 0.83 ± 0.019; NR, 0.927 ± 0.022; Figure 1I,J）.

### 3.2 NR administration protects against Ang ‐–induced BBB dysfunction

**Figure 2:**
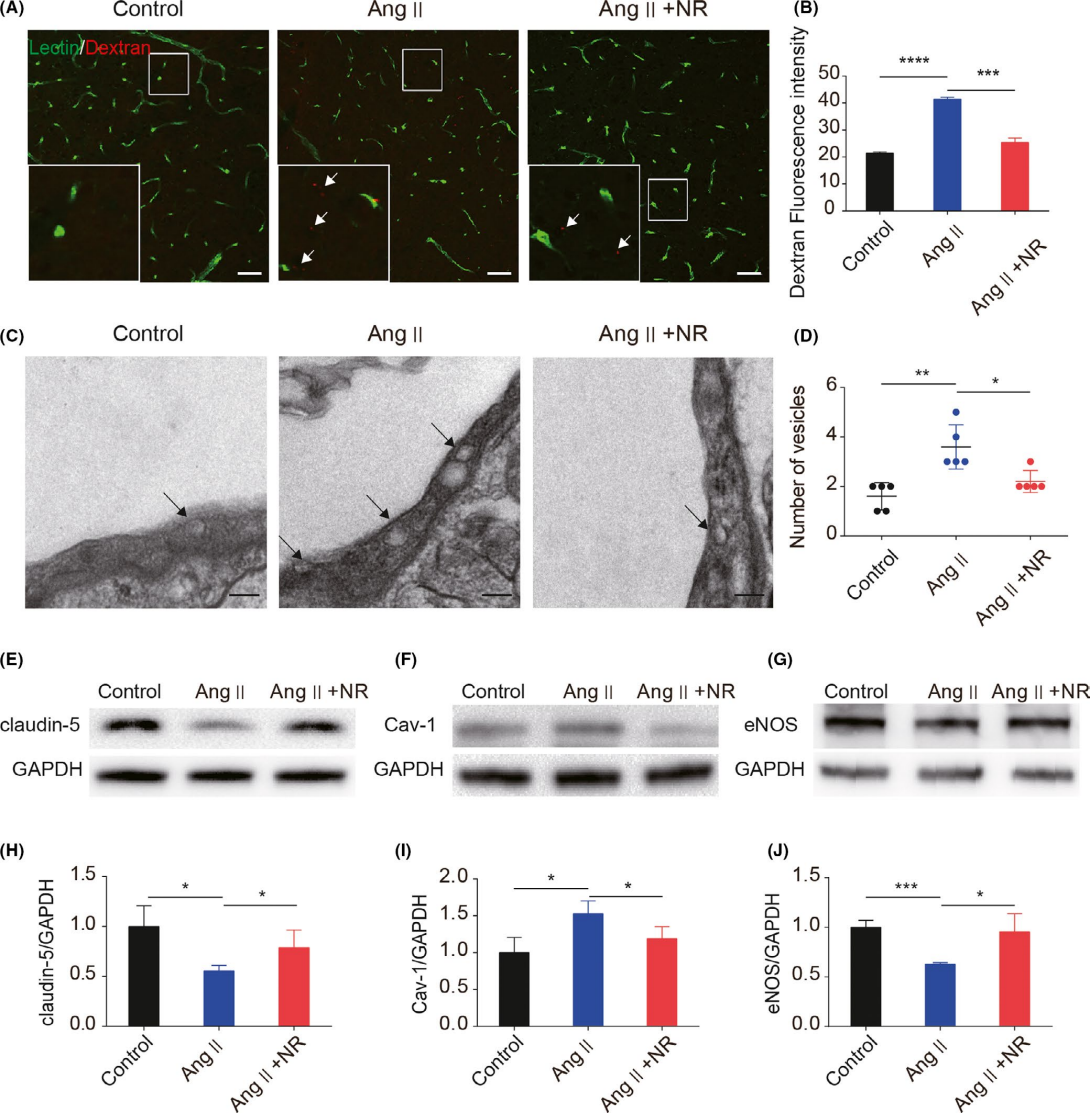
NR administration protects against angiotensin ‐–induced BBB leakage by restoring tight junction proteins and reducing caveolae‐mediated endocytosis. (A‐B) Representative images and the quantification of 3‐kDa dextran fluorescence in mice treated with control, Ang ‐, and NR for 28 d after staining with lectin. The white arrows indicate dextran leakage. (C‐D) Transmission electron microscopy analysis for endocytosis in capillaries. (E, H) Immunoblotting analysis and the quantification of the tight junction protein claudin‐5 in the 3 groups after treatment for 28 d. (F, I) Representative immunoblotting images and the quantification of Cav‐1 in brain tissues from the 3 groups. (G, J) Representative immunoblotting images and the quantification of eNOS expression in brain tissues from the 3 groups. Scale bars: A = 50 µm, C = 2 μm; *P < .05, **P < .01, ***P < .001. Ang: angiotensin ‐, Ang + NR: angiotensin ‐+nicotinamide riboside. The results are presented as the means ± standard deviations (SDs)

Endothelium‐associated BBB dysfunction is considered the driving force behind CSVD.^30^ To test the effect of NR on BBB integrity, FITC‐dextran (3 kDa) was used to detect BBB leakage. NR administration significantly decreased Ang ‐–induced BBB leakage (control, 21.41 ± 0.28; Ang ‐, 41.45 ± 0.42; NR, 25.37 ± 0.97; Figure 2A,B). To explore the mechanism by which BBB integrity was reversed, we first detected the expression of tight junction proteins and found that the expression of claudin‐5 was decreased in the Ang ‐ group compared with the control group. NR administration significantly increased the expression of claudin‐5 (Figure 2E,H). Limited caveolae‐mediated endocytosis is critical for maintaining BBB integrity. The data showed that Cav‐1 expression was remarkably increased in the Ang ‐ group (control, 1.00 ± 0.11; Ang ‐, 1.52 ± 0.099; NR, 1.18 ± 0.095; Figure 2F,I). Additionally, transmission electron microscopy analysis suggested that the number of vesicles was increased in the endothelium (Figure 2C,D). NR administration reduced the Cav‐1 level and vesicle number in capillaries. Furthermore, NR supplementation rescued the eNOS decrease induced by Ang ‐ (control, 1.00 ± 0.040; Ang ‐, 0.62 ± 0.0099; NR, 0.95 ± 0.10; Figure 2G,J). The results above show that NR administration protects against Ang ‐–induced BBB dysfunction in CSVD.

### 3.3 NR administration reduces Ang ‐–induced vascular remodeling

**Figure 3:**
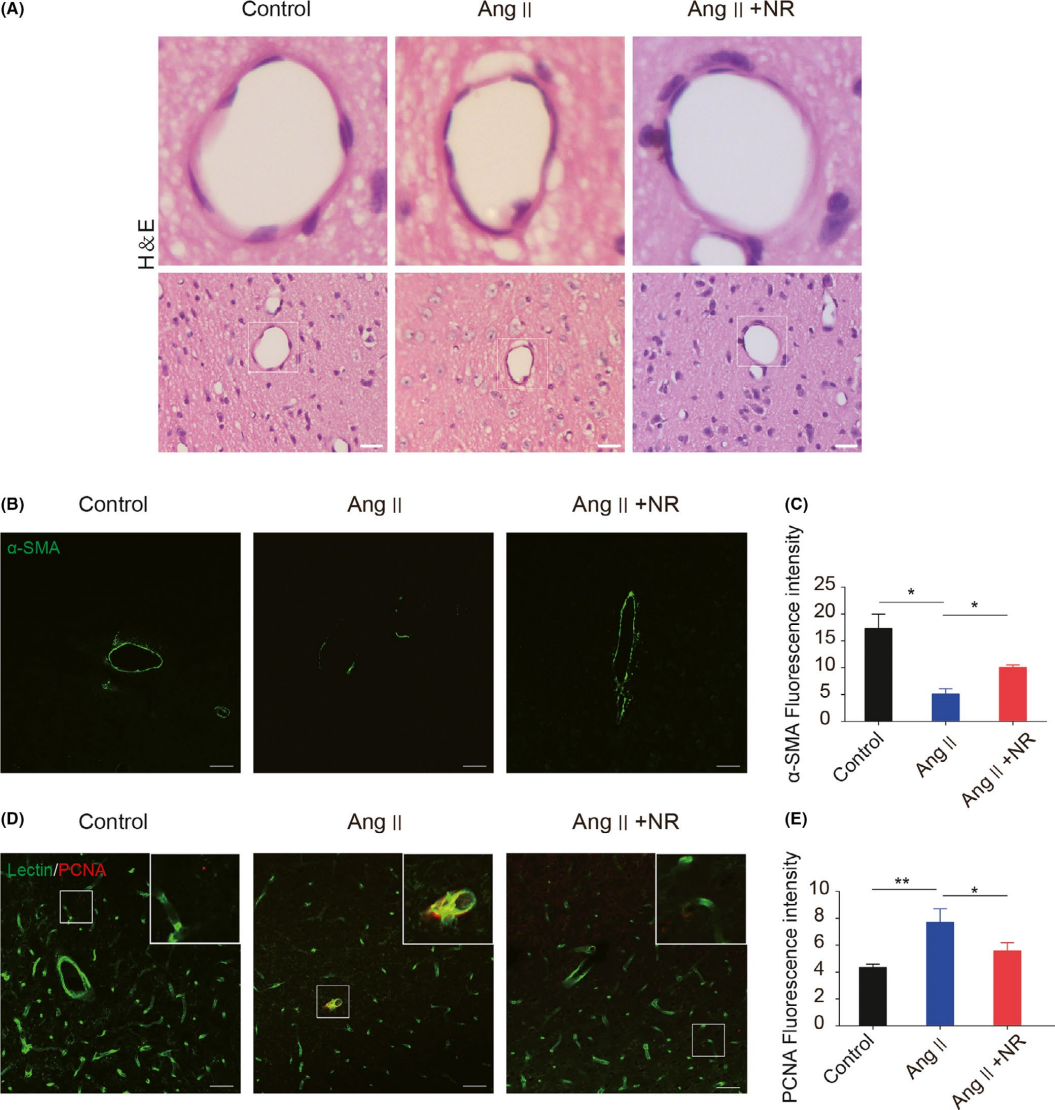
NR administration reduces the angiotensin ‐–induced phenotypic transformation of smooth muscle cells. (A) Representative images of arterioles stained with H＆E in control, Ang ‐–treated, and NR‐treated mice after 28 d. (B‐C) Representative immunofluorescence images and the quantification analysis of α‐SMA in the 3 groups. (D‐E) Representative immunofluorescence images and the quantification analysis of PCNA in the 3 groups after staining with lectin. Scale bars: A = 100 μmol/L, B and D = 50 μm; *P < .05, **P < .01. Ang: angiotensin ‐, Ang + NR: angiotensin ‐+nicotinamide riboside. The results are presented as the means ± standard deviations (SDs)

Arteriole damage has been reported as a pathological change in CSVD. The phenotypic conversion of smooth muscle is one of the major reasons for arteriole damage. Under pathological conditions, especially the activation of the renin‐angiotensin system, smooth muscle cells change from contractile type to proliferative type.^31^, ^32^ Hematoxylin and eosin (H＆E) staining showed that NR administration significantly alleviated vascular wall thickening caused by Ang ‐ (Figure 3A). The Ang ‐ group showed decreased expression of the contractile type marker α‐SMA, and NR administration significantly upregulated the expression of α‐SMA (control, 17.29 ± 2.68; Ang ‐, 5.09 ± 1.00; NR, 10.05 ± 0.47; Figure 3B,C), which was detected by immunofluorescence. Moreover, the immunofluorescence data demonstrated that the expression of the proliferation marker PCNA was significantly increased in the Ang ‐ group, whereas it was normalized by treatment with NR (control, 4.35 ± 0.14; Ang ‐, 7.69 ± 0.58; NR, 5.57 ± 0.35; Figure 3D,E).

### 3.4 NR administration suppresses Ang ‐–induced vascular inflammation

**Figure 4:**
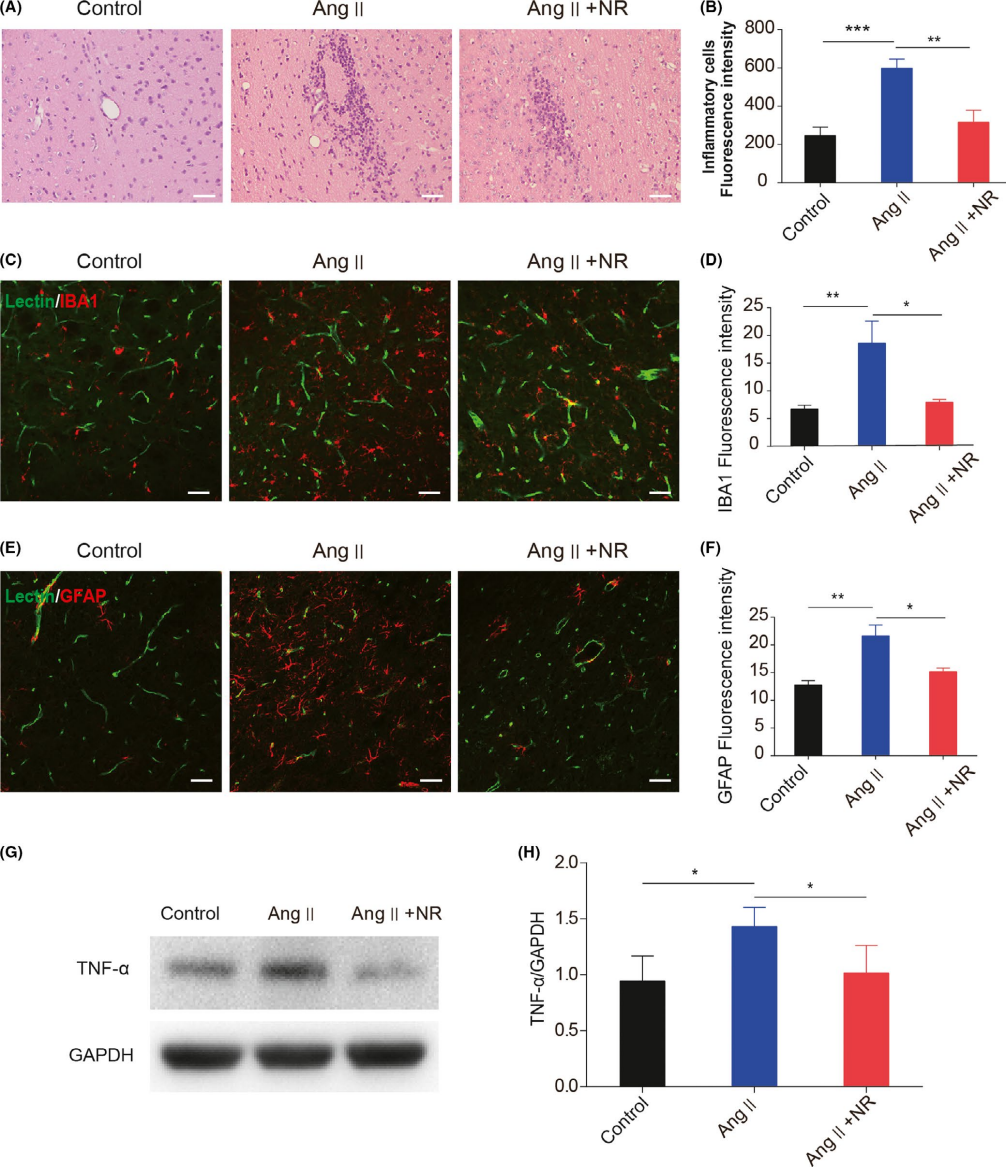
NR administration suppresses the angiotensin ‐–induced excessive activation of microglia and astrocytes. (A‐B) Representative images of inflammatory cells in the brains of control, Ang ‐–treated, and NR‐treated mice after 28 d. (C‐F) Representative immunofluorescence images and the quantification of IBA1 and GFAP after staining with lectin in control, Ang II–treated, and NR‐treated mice. (G‐H) Immunoblotting analysis and the quantification of TNF‐α in the 3 groups. Scale bars A = 100 μmol/L, C and E = 50 μm; *P < .05, **P < .01, ***P < .001. Ang: angiotensin ‐, Ang + NR: angiotensin ‐+nicotinamide riboside. The results are presented as the means ± standard deviations (SDs)

Evidence from numerous articles has suggested a relatively stable association between CSVD and inflammation.^30^ We explored the antiinflammatory effect of NR on CSVD, and H＆E staining showed that NR administration suppressed the infiltration of inflammatory cells caused by Ang ‐ (Figure 4A,B). The data showed that the immunofluorescence intensities of IBA1 and GFAP were enhanced around capillaries and in the brain parenchyma. NR administration significantly suppressed microglial and astrocyte activation compared with that in the Ang ‐ group（control, 6.72 ± 0.38; Ang ‐, 18.58 ± 2.31; NR, 7.95 ± 0.28; Figure 4C‐F）. The increased expression of tumor necrosis factor α (TNF‐α) was also restrained by NR supplementation (control, 0.94 ± 0.12; Ang ‐, 1.43 ± 0.098; NR, 1.01 ± 0.14; Figure 4G,H).

## 4 DISCUSSION

There is a high incidence of CSVD in elderly individuals over the age of 60 years, and CSVD is 6‐10 times more common than large vessel stroke.^4^ Antihypertensive therapy, the most commonly used treatment, cannot efficiently improve all the symptoms of CSVD.^3^ Vascular injury–related pathological changes are the main causes of the poor outcomes of CSVD.^1^ Tarantini et al^17^ reported that supplementation with NAD+ precursor has a protective effect on vascular injury and dementia. In this study, we explored the beneficial effects of NR on Ang ‐–induced CSVD. We found for the first time that NR administration can protect the vasculature and protect against white matter injury and cognitive decline caused by Ang ‐. NR shows the potential to be used as a prospective supplement to antihypertensive therapy for CSVD.

Magnetic resonance imaging has indicated that there is diffuse WMH, which are considered an indicator of dementia and decline in cognitive performance.^33^, ^34^ Cognitive dysfunction is one of the symptoms that cannot be alleviated by antihypertensive therapy.^35^ CSVD patients show slight cognitive changes, such declines in executive functions, attention, and set‐shifting abilities, which can be detected through the Mini‐Mental Status Examination (MMSE).^36^, ^37^ Functional brain imaging, used to reveal the mechanisms of cognitive dysfunction, has suggested that the default mode network (DMN) and frontoparietal control network (FPCN)/ dorsal attention network (DAN) are reduced in CSVD patients.^4^ The mechanism of CSVD has also been evaluated in animal models. Rajani et al^38^ reported that mature oligodendrocytes are decreased, which implies abnormal myelination. In the CNS, MBP and NF200 are the critical components of myelin and axons, and their integrity plays an important role in maintaining the conduction of nerve fiber bundles.^39^ In our study, cognitive decline and myelin degeneration were observed after mice were infused with Ang ‐ for 28 days. Under NR administration, the cognitive function of mice was significantly improved, and the degradation of myelin was repressed. These data indicate that NR protects the cognitive function of mice by attenuating Ang ‐–induced white matter injury, especially the breakdown of axons and myelin.

Higher WMHs and increased BBB permeability are associated with poor functional outcomes in CSVD patients.^40^ The impairment of the BBB predicts cognitive impairment at one year.^41^ Previous studies of BBB permeability in CSVD have focused on tight junction proteins, such as zonula occludens‐1 (ZO‐1), occludin, and claudin‐5.^42^, ^43^ The decreased expression of claudin‐5, the key tight junction protein of the BBB, has been reported in a CSVD model.^38^ In addition, growing evidence has indicated that limited caveolae‐mediated transcytosis maintains BBB integrity in the CNS,^44^ and BBB disruption due to increased vesicle transport has been confirmed in many models.^27^, ^45^ Cav‐1 is a major structural component of caveolae and is mainly expressed in the vessels of the CNS.^46^ Umesalma et al reported that Ang ‐ increases the expression of Cav‐1 in the CNS in hypertension.^25^, ^47^ Andreone et al^45^ reported increased caveolae‐mediated intracellular transport of vesicles can induce BBB leakage. In our study, NR administration not only maintained the expression of claudin‐5 but also inhibited caveolae‐mediated vesicle trafficking, thereby protecting BBB integrity. It was demonstrated for the first time that NR administration restored BBB function by restraining caveolae‐mediated endocytosis and reversing the expression of the tight junction protein claudin‐5 in CSVD.

Animal experiments and clinical evidence have shown that there is a significant correlation between vascular inflammation and CSVD.^30^ BBB destruction leads to the infiltration of inflammatory cells and is the cause of neuroinflammation in CSVD.^48^ The inflammatory response exists throughout the entire process of CSVD.^30^ Inflammation markers, such as fibrinogen, interleukin‐6, and homocysteine, are increased in CSVD patients.^30^ Therefore, inflammation may be the driving force behind CSVD.^1^ Additionally, the CNS inflammation caused by Ang ‐ is extensive. BBB leakage leads to Ang ‐ entering the perivascular space,^49^ where Ang ‐ activates astrocytes and microglia by activating NFкB and signal transducer and activator of transcription protein 3 (STAT3) signaling.^11^, ^50^ Activated glial cells release inflammatory factors that contribute to subsequent neuroinflammation.^51^ In our study, we found that NR administration reduced neuroinflammation and suppressed the activation of microglia and astrocytes by decreasing the expression of TNF‐α in the brain. The antiinflammatory effect of NR may be related to its ability to protect BBB integrity.

## 5 CONCLUSION

Our results demonstrate that NR administration can ameliorate CSVD induced by Ang ‐, including vascular and white matter injury and cognitive decline. The beneficial effects of NR may involve its antiinflammatory ability and the protection of BBB integrity. Further studies are needed to confirm our findings.

## CONFLICT OF INTEREST

The authors declare no conflict of interest.
